# The effects of TeaCrine® and caffeine on endurance and cognitive performance during a simulated match in high-level soccer players

**DOI:** 10.1186/s12970-019-0287-6

**Published:** 2019-04-18

**Authors:** Marissa L. Bello, Alan J. Walker, Bridget A. McFadden, David J. Sanders, Shawn M. Arent

**Affiliations:** IFNH Center for Health and Human Performance, Rutgers University, 61 Dudley Rd, New Brunswick, NJ 08901 USA

**Keywords:** Theacrine, Caffeine, Soccer, Cognitive function, Endurance

## Abstract

**Background:**

Theacrine (1,3,7,9-tetramethyluric-acid) is a pure alkaloid with a similar structure to caffeine and acts comparably as an adenosine receptor antagonist. Early studies have shown non-habituating effects, including increases in energy and focus in response to Teacrine®, the compound containing pure theacrine. The purpose of this study was to determine and compare the effects of Teacrine® and caffeine on cognitive performance and time-to-exhaustion during a simulated soccer game in high-level male and female athletes.

**Methods:**

Male and female soccer players (*N* = 24; M_Age_ = 20.96 ± 2.05y, M_MaleVO2max_ = 55.31 ± 3.39 mL/O_2_/kg, M_FemaleVO2max_ = 50.97 ± 3.90 mL/O_2_/kg) completed a 90-min simulated treadmill soccer match over four randomized sessions (TeaCrine®, caffeine, TeaCrine® + caffeine, placebo). Cognitive testing at halftime and end-of-game including simple reaction time (SRT), choice RT (CRT), and cognitive-load RT with distraction questions (COGRT/COGRT_Wrong_) was performed, with a run time-to-exhaustion (TTE) at 85% VO_2max_ following end-of-game cognitive testing. Session times and pre-exercise nutrition were controlled. RM-MANOVAs with univariate follow-ups were conducted and significance was set at *P* < 0.05.

**Results:**

TTE trended towards significance in TeaCrine® and TeaCrine® + caffeine conditions compared to placebo (*P* < 0.052). A condition main effect (*P* < 0.05) occurred with faster CRT in caffeine and TeaCrine® + caffeine compared to placebo. COGRT_Wrong_ showed a significant time main effect, with better accuracy at end-of-game compared to halftime (*P* < 0.05). A time x condition interaction in SRT (*P* < 0.05) showed placebo improved from halftime to end-of-game.

**Conclusions:**

The 27–38% improvements in TTE reflect increased performance capacity that may have important implications for overtime scenarios. These findings suggest TeaCrine® favorably impacts endurance and the combination with caffeine provides greater benefits on cognitive function than either supplement independently.

## Background

Caffeine (1,3,7-trimethylxanthine) is one of the most commonly researched supplements and has considerable support for ergogenic effects over a wide range of sports [1, 2] with moderate-to-large doses (3–6 mg/kg BW or ~ 200–400 mg). Caffeine functions as a competitive inhibitor of adenosine, regulating sleep/wake cycles by binding to adenosine receptors to block their actions while increasing concentrations of neurotransmitters such as dopamine and serotonin to mediate concentration, mood, and fatigue [3–7]. Additionally, this inhibition of adenosine receptors alters the autonomic nervous system, subsequently increasing systolic blood pressure and heart rate, with further augmentation of this response under exercise conditions [8–10]. Furthermore, there are effects on cognitive function via the three networks of executive attention, orienting, and alerting, with moderate doses shown to induce cognitive performance improvements in soccer players and fencers [11–14]. In addition to psychophysiological effects, caffeine has been shown to produce notable ergogenic effects on aerobic capacity. Much of the evidence for this effect pertains to improved time trial performance in cyclists and running time-to-exhaustion in distance runners when consumed in doses from 2 to 6 and 3–9 mg/kg BW, respectively [15–17]. While similar benefits have been suggested for anaerobic performance, the results are mixed with some studies showing improvements in mean sprint times in swimmers and increases in peak power measured via Wingate tests, while other studies have shown no significant changes in 1RM strength [18–22]. However, it is important to note that the research investigating sports with both aerobic and anaerobic components has primarily used energy drinks to explore the effects of caffeine on performance outcomes, which may confound interpretations of caffeine’s ergogenic effects due to potential compound interactions of ingredients within the energy drinks [23–25].

Although caffeine has been shown to improve several aspects of performance, there are several undesirable side effects potentially associated with it, including the increases in cardiovascular responses, habituation from chronic use, and timing effects that should be taken into consideration [4, 11, 26]. A recently developed compound similar to caffeine, theacrine (1,3,7,9-tetramethyluric acid), may hold some promise given that it has a longer onset of action at approximately 2 h and has been shown to increase mood and subjective measures of cognitive function with no adverse side effects or habituation [27–29]. Theacrine appears to operate similarly to caffeine as an adenosine receptor antagonist, so it can be hypothesized that the use of TeaCrine® might mimic and provide longer-lasting effects than caffeine, without the sharp decline in effectiveness that usually occurs as the concentration of caffeine decreases in the body [28, 30–32]. Currently, there is only one study that has investigated the effects of theacrine independently on subjective measures of mental well-being including such things as energy, focus, and fatigue, with significant improvements noted [29]. An additional study has tested the effects on muscular strength and endurance in resistance trained males, with few significant beneficial effects on strength training and endurance during bench and leg presses [30].

Due to their similar mechanisms and varying half-lives, a majority of the research has focused on the combination of caffeine and theacrine to produce fast, yet prolonged ergogenic effects. Studies have shown no adverse effects on heart rate or blood pressure compared to either supplement independently, suggesting this combination is safe to be administered in doses of 125 mg theacrine/150 mg caffeine [27, 31]. A proprietary blend of caffeine and theacrine (TheaTrim) was shown to have no significant impact on subjective measures of cognitive function, although there were significant effects noted for increases in subjective feelings of focus and energy [31]. However, due to the fact that the quantity of theacrine in this combination was undisclosed, the results should be interpreted with caution. Given the limited information and mixed results, further investigation into the potential benefits of theacrine and threacrine + caffeine in sports performance is warranted.

The efficacy of these supplements in athletic settings may largely depend on the physical and cognitive demands of the sport. For example, soccer is highly aerobic but also includes a mix of anaerobic power and cognitive load, with all three contributing to predict a player’s performance and success [33–36]. Reported ranges for total distance in a match is between 8 and 13 km, with changes in speed every six seconds on average, indicating the intermittent nature and varying demands of a soccer match [36–38]. Furthermore, the utilization of executive function has been found to be a predictor of success in soccer [33, 34, 39, 40] and soccer players have demonstrated the ability to reallocate resources under temporal and spatial constraints. Therefore, executive function may play a large role in match play, particularly under fatiguing conditions [35, 41, 42]. Supplements such as caffeine and/or theacrine may enhance athletic performance by improving a player’s work capacity, as well as by mitigating the effects of fatigue on cognitive function. Limited research exists observing the differences between caffeine and theacrine in the area of sport performance, as well as the effects of the combination of the two supplements. Use of an ergogenic aid that could enhance cognitive ability and reduce fatigue without a “crash” afterwards or habituation effect could improve a player’s ability to sustain a higher level of play for a longer period of time. The purpose of this study was to determine the effects of TeaCrine® and caffeine compared to placebo on various measures of cognitive performance under fatiguing conditions of a simulated match load in high-level male and female soccer players. Secondary purposes were to determine whether TeaCrine® and caffeine in combination have a synergistic effect, as well as the impact on time-to-exhaustion in an overtime scenario.

## Methods

A within-subjects, placebo-controlled, double-blind design was used to determine the effects of caffeine and TeaCrine® on performance. Subjects completed four test sessions in randomized order after ingesting either 275 mg placebo (PL), 275 mg TeaCrine® (TCr), 275 mg caffeine (Caf), or a 125/150 mg combination of TeaCrine® and caffeine (TCr + Caf) 30 min prior to exercise. This time-frame was used in order to allow the caffeine and Teacrine® to be absorbed and achieve peak concentration through the middle of the test session. Absolute dosing was chosen due to previous research that used similar absolute amounts of TeaCrine® in addition to practical supplementation strategies. All supplements were consumed as a single capsule. PL capsules were filled with 275 mg of cellulose. Participants completed all sessions at the same time of day (within 1 h) and were instructed to abstain from vigorous exercise and caffeine consumption for 24 h prior to each session. Experimental sessions were separated by a minimum of at least 48 h.

### Subjects

Male (*n* = 12) and female (*n* = 15) Division I and professional soccer players were recruited for this study. All subjects were highly trained, participating in soccer-related activities a minimum of 5 days per week at the time of the study. Subjects were excluded if they had any injuries that would prevent them from completing the protocol, had a history of caffeine sensitivity, drank more than the equivalent of four cups of percolated coffee per day, or currently took OTC products containing pseudoephedrine or other stimulants. All subjects read and signed an informed consent form and the study was approved by the Rutgers University Institutional Review Board. Three subjects (two males, 1 female) were excluded from the statistical analysis due to noncompliance. Subject characteristics are presented in Table 1.

**Table 1:** Subject Demographics

Variable	Males (*n* = 10)	Females (*n* = 14)
Age (yrs)	21.8±2.53	19.65±3.62
Height (cm)	178.6±6.88	165.2±9.78
Weight (kg)	74.7±8.67	65.76±8.01
Body Fat (%)	10.76±3.48	19.64±3.62
VO_2max_ (mL/kg/min)	55.31±3.39	50.97±3.90

Data represented as mean±standard deviation

### Fitness testing and familiarization

Subjects were instructed to arrive at the Rutgers Center for Health and Human Performance (CHHP) euhydrated, 2 h fasted, and having refrained from exercise 24 h prior to testing. Body weight (BW), body composition (%BF), lean body mass (LBM), and fat mass (FM) were measured using air-displacement plethysmography (BodPod, COSMED, Concord, CA, USA) according to manufacturer’s guidelines.

Participants completed a dynamic warm-up prior to performing a VO_2max_ test. VO_2max_ was assessed using a treadmill graded exercise test. The test consisted of a constant 2.0% incline grade with speed increases every 2 min until exhaustion [43]. Males began the protocol at 7.9 km/h, while females began at 6.4 km/h. Speed increases for subsequent stages of the protocol were 10.0, 11.7, 13.7, 15.6, 17.1, 18.2, 19.8, and 21.1 km/h. Rating of perceived exertion (RPE) was obtained at the end of each stage [44].

Ventilatory, metabolic, and cardiovascular responses were continuously monitored using direct gas exchange with breath by breath sampling using a Quark CPET metabolic cart (COSMED, Concord, CA, USA). The gas analyzers and spirometer were calibrated prior to each test according to manufacturer’s guidelines [45]. Heart rate (HR) was continuously monitored using the Polar H7 HR transmitter (Polar Electro Co., Woodbury, NY, USA).

Participants were familiarized with the tasks to be performed in each session. The Dynavision D2™ Reaction Board was used for cognitive testing (Dynavision International LLC, Chester, OH, USA). Three familiarization rounds using the same procedures to be employed during actual testing were performed on the reaction board during this initial visit [46]. Three 1-min trials, with 1-min rest periods, were used to test reaction time. The first trial tested simple reaction time (SRT) via the pressing of a light stimulus that appeared in a random location on the board. SRT was scored as time for participants to press the stimulus averaged over the course of the minute. The second trial tested choice reaction time and score (CRT and CRT_Score_), using a red and green stimulus to designate “go/no-go”, where the red stimulus was “go” and the green stimulus was “no-go”. Similarly to SRT, CRT was scored using average RT of pressing the red stimulus, and CRT_Score_ was scaled based on number of red targets pressed and green targets avoided, calculated as “total number of red hit plus green avoided” divided by the total number of targets that appeared during the minute.

The last trial tested cognitive load (i.e., complex) reaction time (COGRT) and score (COGRT_Score_) with the “go/no-go” light stimulus and cognitive load tasks (COGRT_Wrong_), which consisted of a series of either four letters, which the subject had to repeat aloud in the correct order, or a simple math problem, which the subject had to answer correctly. COGRT was scored similarly to SRT and CRT for average RT of pressing the red stimulus. COGRT_Score_ was scaled based on number of red targets pressed and green targets avoided, calculated as “total number of red hit plus green avoided” divided by the total number of targets that appeared during the minute. The “load” tasks were shown every 3 s and remained on the screen for 1 s for a total of 20 questions per block. Subjects were able to answer until the next question appeared, and answers were recorded as correct, incorrect, or no answer. These questions were scored as a percentage answered wrong (CRT_Wrong_), with incorrect and no answers contributing to this score. One full round of testing included one trial of each reaction time task separated with 1 min recovery [46].

### Procedures

Participants recorded their 24 h dietary intake prior to their initial performance testing session. For all subsequent sessions, subjects recorded an additional diet log, which was compared by a study member to ensure an identical diet. If subjects were deemed to deviate from their standard diet, they were rescheduled. Participants wore a Polar H7 HR transmitter synced to a Polar V800 watch to monitor HR throughout the session. Upon arriving at the CHHP, participants consumed the session-assigned capsule with water, and after a 15-min period of quiet rest, began a 15-min warm-up. A general warm-up of 5 min of aerobic activity was performed on a treadmill at a self-selected pace. Next, a dynamic warm-up was performed consisting of high knees, butt kicks, lunges, lunge with a rotation of the upper body, power skips, power skips into a lunge, straight leg kicks, hamstring walk-outs, and lateral squats.

**Fig. 1:**
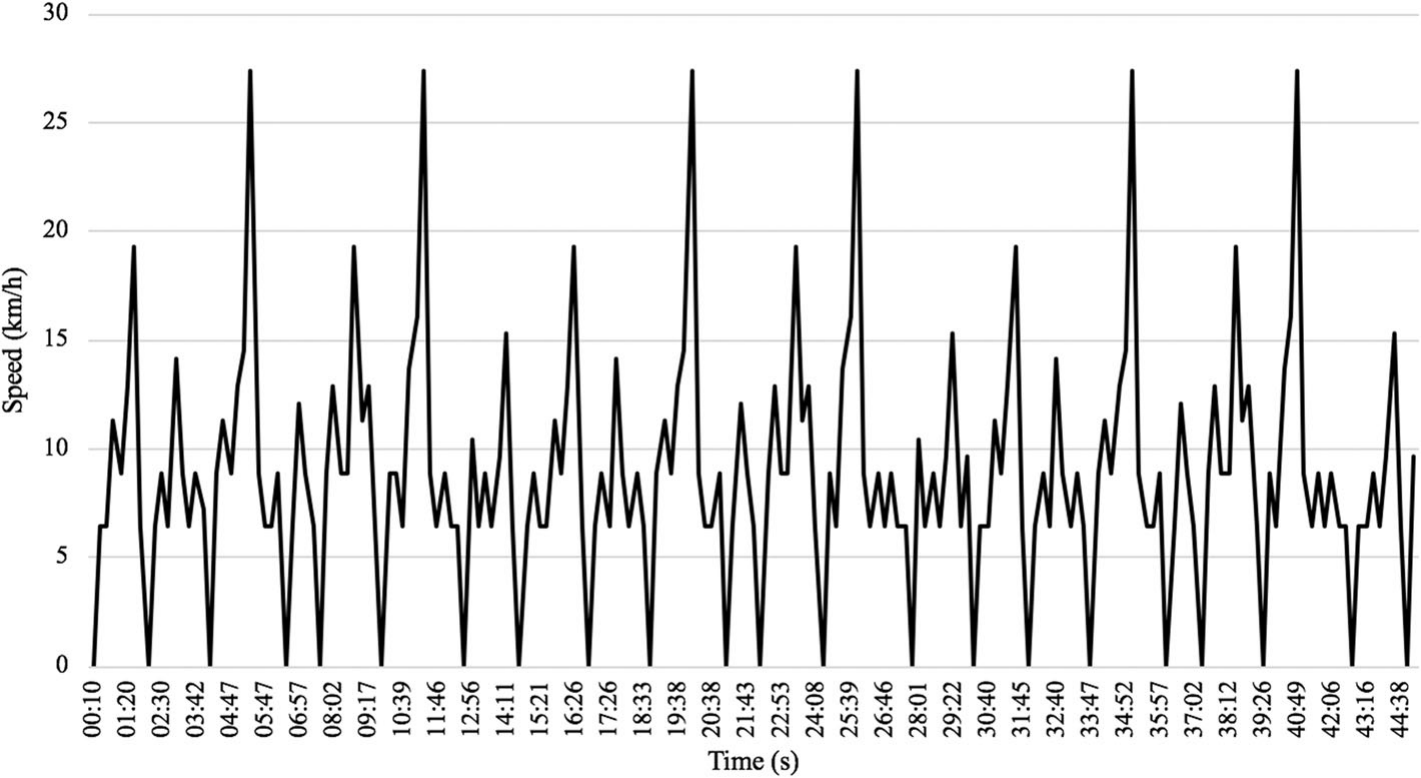
Simulated game protocol

Following the warm-up, subjects proceeded into the testing room to complete a 90-min simulated soccer game protocol on a high-speed treadmill (HPCosmos T170, COSMED, Concord, CA, USA). The simulated game also included a 15-min half-time. The protocol was comprised of the varying exercise intensities characteristic of match-play (e.g., stationary, walking, jogging, running, and sprinting; see Table 2). The speed zones and relative time spent in each were based on research in high-level male players [47]. The higher speeds for the female players were scaled accordingly (~ 5–12% slower) based on GPS game data obtained from Division I college players at Rutgers University (Fig. 1). The first and second half were identical (Fig. 1), with a total game distance of 12.86 and 12.7 km for males and females, respectively. RPE was assessed at each 15-min interval of the protocol. Testing environment was controlled for each session.

**Table 2:** Activity Profile

Mode	Km/h	% Time	Grade
Standing	0	10.11%	2.00%
Walking	<8.05	33.30%	2.00%
Jogging	8.06-9.66	32.59%	2.00%
Running	9.67-14.48	16.11%	2.00%
Intense Running	14.49-22.53	6.67%	2.00%
Sprinting	>22.53	1.22%	2.00%

Following the conclusion of the first half, subjects performed one full round of cognitive testing using the same procedure as described for the familiarization session, and the remaining time was used as a rest period for a total time of 15 min. After completion of the second half, subjects performed a second full round of cognitive testing. Following completion of the final cognitive test, which was consistent with the 5-min rest period between the end of regulation and beginning of overtime in an actual match, subjects were placed back on the treadmill and ran at a speed corresponding to 85% of their VO_2max_. Subjects were instructed to run at this speed until volitional fatigue, with no motivation from the testers. This was recorded as their run time-to-exhaustion (TTE). The treadmill clock and speed were covered so the participants were blinded to time and distance.

### Statistical analysis

Descriptive statistics (mean ± SD) were used to quantify subjects’ physical characteristics. RM MANOVAs with univariate follow-ups were used to determine differences among conditions for SRT, CRT, and COGRT. Univariate follow-ups and simple contrasts (using PL as the comparison condition) were performed following significant multivariate effects. Separate RM ANOVAs were used to determine differences in TTE for the “overtime” runs, RPE, and HR for each session. Results were considered statistically significant when the probability of a type I error was less than 0.05 (*P* < 0.05).

For each univariate analysis, the assumption of sphericity was tested using an examination of the Huynh–Feldt (H–F) epsilon for the general model. If this statistic was greater than 0.75, sphericity was considered to have been met, and the unadjusted univariate statistic was used. If epsilon was less than 0.75, a violation of the assumption of sphericity was considered to have occurred, and the H–F adjusted statistic was used to determine significance. Effect sizes (ES) were calculated in order to compare magnitude of changes for each experimental condition compared to PL using Hedges’ *g* formula for ES computation. Additionally, ES was calculated for changes within each condition from halftime to end-of-game. This ES computation was used for all variables, with a positive ES representing better or faster results, and a negative ES representing slower or worse results.

## Results

There were no main effects or interactions for sex for any of the variables (*P* > 0.20). Because of this, data were collapsed across sex for all remaining analyses. There was a significant multivariate effect for the conditions (*P* = 0.025), therefore univariate follow-ups for each variable were conducted. All cognitive results are presented in Table 3.

**Table 3:** Cognitive Measures

Measure	Condition	Halftime Mean±SD	Hafltime ES	End-of-Game Mean±SD	End-of-Game ES
SRT (s)	PL	0.647±0.059		0.631±0.047	
	TCR	0.645±0.054	0.04	0.659±0.070*	-0.47
	CAF	0.629±0.057*	0.31	0.647±0.053	-0.32
	TCR+CAF	0.635±0.058	0.21	0.647±0.066	-0.28
CRT (s)	PL	0.614±0.069		0.602±0.059	
	TCR	0.607±0.067	0.10	0.612±0.074	-0.15
	CAF	0.593±0.054*	0.34	0.597±0.060	0.08
	TCR+CAF	0.592±0.057*	0.35	0.588±0.060*	0.24
CRT-Score	PL	98.652 ±0.394		98.678±1.611	
	TCR	97.383±0.512*	-2.78	98.287±1.967	-0.22
	CAF	98.848±1.302	0.20	99.030±1.619	0.22
	TCR+CAF	98.548±1.608	-0.09	98.370±1.748	-0.18
COGRT (s)	PL	0.676±0.059		0.678±0.055	
	TCR	0.679±0.062	-0.03	0.678±0.060	0.00
	CAF	0.658±0.047*	0.34	0.677±0.053	0.019
	TCR+CAF	0.665±0.054	0.19	0.667±0.055	0.20
COGRT-Score	PL	89.470±7.376		88.674±5.800	
	TCR	89.557±6.268	0.013	89.030±6.364	0.06
	CAF	90.700±5.821	0.019	90.048±5.807	0.24
	TCR+CAF	89.474±5.501	0.001	90.191±6.537	0.25
COGRT-Wrong (%)	PL	18.48±12.56		16.30±10.25	
	TCR	18.70±10.89	-0.02	16.96±12.77	-0.06
	CAF	19.44±12.46	-0.08	14.13±12.12	0.19
	TCR+CAF	18.91±13.81	-0.03	17.17±11.66	-0.08

* Denotes significant (*P* <0.05) compared to PL

### Simple reaction time

There was a significant time main effect (*P* = 0.031) for SRT from halftime to end-of-game. However, a condition x time interaction (*P* = 0.022) revealed that PL improved from halftime to end-of-game for SRT while all other conditions showed slower RT from halftime to end-of-game (0.647 ± 0.059 vs 0.631 ± 0.047 s, ES_PL_ = 0.27; 0.645 ± 0.053 s vs 0.659 ± 0.070 s, ES_TCr_ = − 0.09; 0.629 ± 0.057 s vs 0.647 ± 0.054 s, ES_Caf_ = − 0.30; 0.635 ± 0.058 s vs 0.647 ± 0.066 s, ES_TCr + Caf_ = − 0.22).

Simple contrasts at halftime showed significant differences between conditions. Caf was faster at halftime when compared to PL (0.629 ± 0.057 s vs 0.647 ± 0.059, *P* = 0.032, ES_Caf_ = 0.31). There were also significant differences between conditions at end-of-game due to a slower SRT for TCr compared to PL (0.659 ± 0.069 vs 0.631 ± 0.047 s, *P* = 0.017, ES = -0.47). There were no other condition differences compared to PL at end-of-game.

### Choice Reaction Time & Score

There was a significant condition main effect for CRT (*P* = 0.003). Univariate follow-ups indicated Caf and TCr + Caf were faster compared to PL (*P* = 0.034 and *P* = 0.000, respectively). There was a condition effect for CRT_Score_ (*P* = 0.008). Univariate follow-ups showed worse scores for TCr compared with PL (*P* = 0.014).

Simple contrasts at halftime showed significant condition differences. Caf produced faster CRT compared to PL (0.593 ± 0.054 s vs 0.614 ± 0.069 s, *P* = 0.012, ES = 0.34). TCr + Caf produced faster CRT compared to PL (0.592 ± 0.057 s vs 0.614 ± 0.069 s, *P* = 0.001, ES =0.35), with a magnitude of effect similar to that for Caf. TCr had worse CRT_Score_ when compared to PL (97.383 ± 0.512 vs 98.652 ± 0.394, *P* = 0.005, ES = -2.78). There were also significant differences between conditions at end-of-game for CRT, with TCr + Caf faster compared to PL, though this effect was small (0.602 ± 0.059 vs 0.588 ± 0.060, *P* = 0.029, ES = 0.24). No other condition differences were noted.

### Cognitive load reaction time, score, & wrong answers

No main effects were seen for COGRT (*P* > 0.13) or COGRT_Score_ (*P* > 0.2), but there was a significant time main effect for COGRT_Wrong_ (*P* = 0.042). Univariate follow-ups indicated greater accuracy at end-of-game compared to halftime across conditions (*P* = 0.037).

Planned simple contrasts at halftime revealed significant differences between conditions for COGRT, with no other condition effects for any other measures. Caf presented faster COGRT when compared to PL (0.658 ± 0.047 s vs 0.676 ± 0.059 s, *P* = 0.049, ES = 0.34). There were no differences compared to PL at end-of-game for any measures (*P* > 0.1).

### Running time-to-exhaustion

**Fig. 2:**
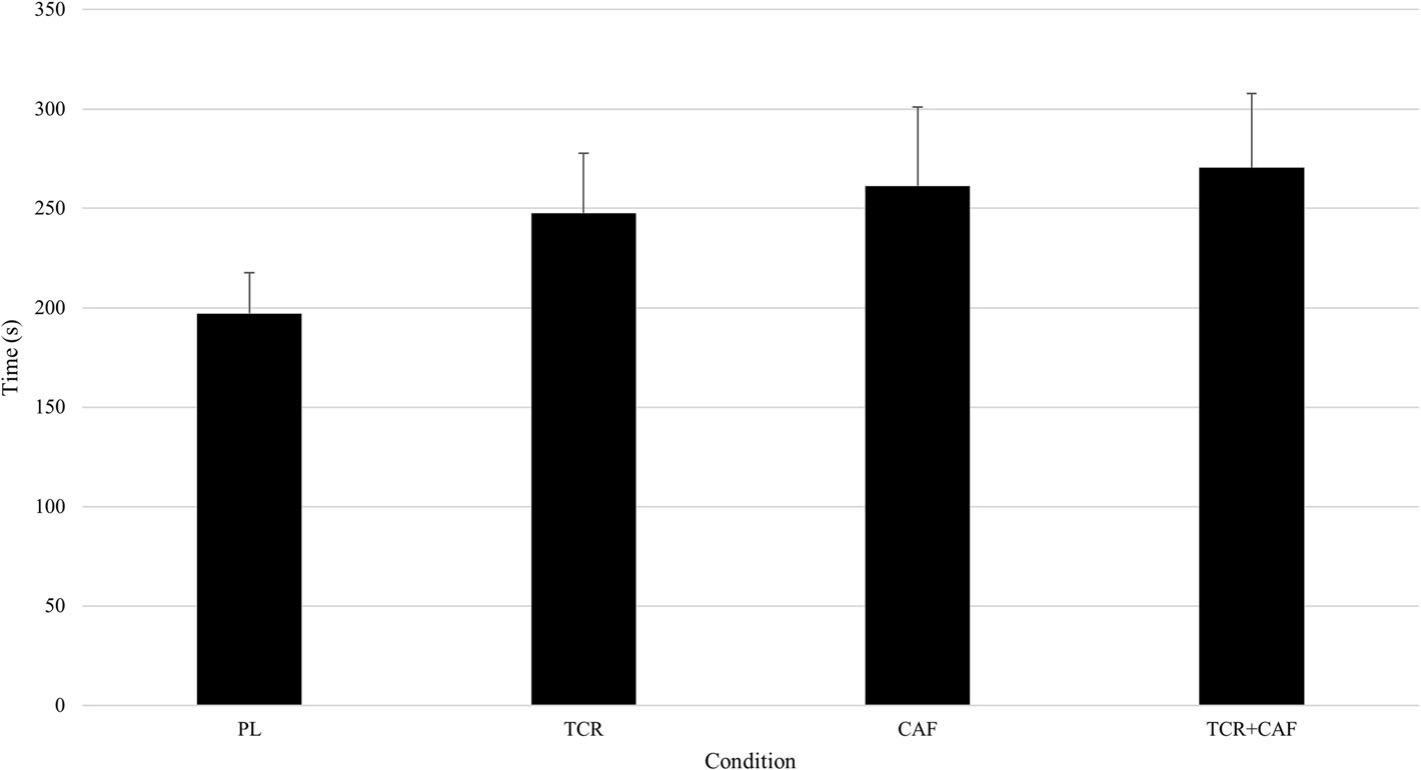
Running time-to-exhaustion (TTE)

There was a trend toward improvements in TTE in all conditions when compared to placebo (TTE_PL_ = 194.1 ± 96.9 s). TCr presented an average increase of 27% (TTE_TCr_ = 245.9 ± 142.3 s, ES_TCr_ = 0.43, *P* = 0.052). Caf increased on average 32% (TTE_Caf_ = 255.4 ± 189.1 s, ES_Caf_ = 0.41, *P* = 0.139), while TCr + Caf showed an average increase of 38% (TTE_TCr + Caf_ = 267.0 ± 175.7 s, ES_TCr + Caf_ = 0.51, *P* = 0.051). These data are shown in Fig. 2. Over 70% of subjects had their longest TTE in the TCr + Caf (45.8%) or Caf (25%) conditions, with 12.5% having the longest TTE in the PL.

### Heart rate

HR was measured as a mean of first half (H1), second half (H2), and overtime (OT; reflected as TTE). There was no significant condition effect. There was a significant time main effect across the simulated soccer protocol, with an increase in HR across all time points (H1 to OT 160.642 ± 9.447 vs 178.260 ± 11.462 bpm, *P* = 0.000, ES = 1.86).

### Rating of perceived exertion

Taken in 15-min intervals, there were six time points for RPE. There was a significant time main effect, with an increase in RPE from T1 to T6 (11.174 ± 1.573 vs 14.690 ± 1.981, *P* = 0.000, ES = 2.23). While there were no significant differences shown between conditions, average RPE across all time points showed a small-to-moderate effect of lower RPE in Caf and TCr + Caf and a trivial effect in TCr compared to PL (ES_Caf_ = − 0.44, *P* = 0.004; ES_TCr + Caf_ = − 0.33, *P* = 0.194; ES_TCr_ = − 0.12, *P* = 0.282).

## Discussion

The primary results of this study indicate that, compared to PL, 275 mg of Caf or a combination of 150 mg Caf with 125 mg TCr produce some modest cognitive benefits, particularly following the first half of the simulated soccer match. These benefits were not seen with ingestion of 275 mg of TCr alone, which was similar to or slightly worse than PL. However, the TTE results suggest notable trends for physical improvement compared to PL for TCr, Caf, and TCr + Caf. The largest effect was seen in the TCr + Caf condition, supporting the possibility that there is a synergistic effect for these supplements, particularly given that the dose of each in combination was less than that given independently.

Similar to previous findings [31], TCr did not significantly improve cognitive measures of performance which could be due to a variety of influences, particularly the timing of testing, as previous research has shown supplement uptake and usage to be influenced by time of day [48]. Although time of day for testing was matched for all visits for each subject, standardizing the session times of different subjects was not logistically feasible. While diet was not controlled between subjects, the within-subject design allowed for controlling diet within each participant, maintaining an identical diet prior to their respective sessions. In addition to the timing of testing, the timing of each supplement may also play an important role in the RT results. These results revealed that all conditions containing Caf showed better cognitive performance at halftime when compared to end-of-game. This aligns with the literature on timing for peak concentration of Caf at 1-h following consumption [12]. Additionally, while the proposed peak concentration of TCr is approximately 2 h, it may occur later than anticipated which potentially explains the lack of beneficial effects for cognition and the relatively smaller benefit for TTE.

The training background of players may also contribute to cognitive management by enhancing processing speed and attention, which may potentially explain the faster SRT at end-of-game in PL. It has been suggested that well-trained individuals can mitigate decreases in cognitive performance under fatiguing conditions [33, 34, 49, 50]. Considering the high level of skill in the athletes used, it is likely that this phenomenon may have contributed to the improvements seen in the PL condition. However, the level of athlete recruited for this study should be noted as a strength in comparison to previous research on Caf or TCr in recreationally-trained participants. The aforementioned training effect may also explain the fewer incorrect responses across all conditions at end-of-game compared to halftime. While there were no significant main effects for COGRT in the current study, the improvements in COGRT_Wrong_ agrees with previous research showing a significant increase in the number of correct responses in a visual vigilance task [51]. Players may allocate resources to maintain performance toward the end of the match when skill and quick decision-making are critical determinants to the outcome.

The increases in TTE across all conditions compared to PL suggests that players can maintain a higher level of performance at later stages in a match with consumption of Caf and TCr. These results trended towards statistical significance (*P* < 0.052), but given the magnitude of differences, the clinical significance may be reflected in implications for late game or overtime scenarios in matches. Although TCr and Caf each had similar magnitudes of positive effects on TTE compared to PL (27 and 32%, respectively), it appears that the combination may have been even more impactful given the 38% increase in TTE. These improvements are supported by previous studies that demonstrated an ergogenic effect of Caf for producing increases in endurance performance in trained cyclists and distance runners following consumption of 2 and 3 mg/kg BW, respectively [4, 17, 52]. With 45.8% of subjects having their longest TTE in TCr + Caf, the effect of the combination condition appears fairly robust. Recent research [53] has also identified potential genetic influences on responsiveness to Caf, though examination of this was beyond the scope of the current study.

It should be noted that a simulated protocol in a lab setting cannot replicate the competitive aspect of a match, particularly during TTE which would coincide with an overtime period. No additional motivation was provided to any subject during this part of the protocol to ensure consistency across conditions, and all subjects were blinded to the treadmill display. This allowed for adequate control of extrinsic factors similarly across conditions, and the results demonstrate consistency in the conditions that produced longer TTE. The TTE results may also be partially explained by a subjective decrease in perceived exertion in these conditions. A meta-analysis on the ergogenic effects of Caf support this notion, showing up to 29% of variance in performance improvements being accounted for by decreases in RPE [52, 54]. Additionally, Caf’s effect on endurance has also been shown to have a glycogen sparing effect through increasing plasma free fatty acids and the rate of lipid metabolism [55, 56]. Additional research needs to be conducted on the mechanistic side of TCr to determine if there is a similar influence on the mobilization of substrates [29], as well as its effects as a dopamine agonist [32].

It is worth noting that a main strength of this study was the simulated soccer protocol, which appears to be a valid tool in replicating the physiological demands of a soccer match. With this intermittent protocol, the HR response was consistent with that of previous studies that demonstrated an average HR ranging from 155 to 172 bpm during a 90-min match, with expected increases in HR between each 45-min half [37, 57]. This may have future application for laboratory-based studies using soccer players. Though it is impossible to fully mimic the competitive demands of a soccer match, it would appear that this protocol was able to simulate the physiological load. This may hold particular value for future research in this area when facilities are not readily available to run other simulated soccer tests, such as the Copenhagen Soccer Test [58]. Additionally, given the lack of sex effects in the current study, it would appear that the modifications to the treadmill protocol speeds accounted for male and female player differences. Additional strengths of this study protocol were the varying levels of difficulty in assessing RT using CRT and COGRT in addition to SRT. Using different tests allowed testing for several aspects of cognitive ability and increase the practicality of the protocol.

When taking the overall cognition and endurance effects into account, it appears that the combination of TCr + Caf was the most beneficial in terms of increasing and maintaining energy, concentration, and level of performance. These results would also suggest the benefits of the combined TCr + Caf supplementation may due to the differences in timing of peak concentration, possibly due to an overlap in the concentration curves of the supplements as shown in previous research [31]. Additionally, the overall improvements in RT and TTE from TCr + Caf may mitigate impaired CNS activity that has been demonstrated in previous research following a 90-min match [33, 34, 59].

In addition to the peak timing differences, there is also the combination of the pharmacology of TCr and Caf to consider. A potential mechanism to explain the observed improvements may be TCr exposure, as a co-ingestion of Caf and TCr has been shown to significantly alter TCr disposition [27]. This results in increased bioavailability and enhanced TCr exposure parameters, including area under the curve plasma concentration and maximum concentration [27]. In the absence of changes in the half-life, the increase peak concentration and area under the curve signify prolonged effects when co-administered with Caf, with no further effects on Caf parameters [27], supporting the notion that timing of supplements may have played a role in the RT data. Furthermore, this may provide a possible explanation for the almost 40% increase in TTE with TCr + Caf. Further research is needed to determine appropriate dosing strategies to optimize the potential benefits of combined Caf and TCr. It should be noted that while previous research has focused on relative dosing strategies, an absolute dose is more consistent with administration methods in this population, and the lack of sex effects supports this notion that an absolute dose elicits similar results.

## Conclusions

This is the first study to our knowledge that has investigated the cognitive and physical performance effects of TCr and TCr + Caf in power-endurance athletes. The combination of TCr + Caf may provide some modest cognitive benefits during complex decision making, potentially due to overlapping peak concentrations or enhanced bioavailability. Similar benefits with a trend towards statistical significance were also seen for TTE, which may have practical importance for extra-time scenarios during matches. Interestingly, the improved cognitive accuracy at end-of-game in all conditions may indicate a training effect in highly skilled players for allocation of resources.

## Abbreviations

%BF: Body composition
BW: Bodyweight
Caf: 275 mg caffeine
COGRT: Cognitive-load reaction time
COGRT_Score_: Cognitive-load score
COGRT_Wrong_: Cognitive-load wrong answers
CRT: Choice reaction time
CRT_Score_: Choice score
ES: Effect size
FM: Fat mass
H1: First half
H2: Second half
HR: Heart rate
LBM: Lean body mass
OT: Overtime reflected as TTE
PL: Placebo
RPE: Rating of perceived exertion
SRT: Simple reaction time
TCr + Caf: 125 mg TeaCrine® + 150 mg caffeine
TCr: 275 mg TeaCrine® or Theacrine
TTE: Run time-to-exhaustion
